# A Model of Butyrate Activity and Resistance in CRC

**DOI:** 10.1111/jcmm.70656

**Published:** 2025-06-12

**Authors:** Michael Bordonaro

**Affiliations:** ^1^ Department of Medical Education Geisinger Commonwealth School of Medicine Scranton Pennsylvania USA

**Keywords:** canonical Wnt signalling, CBP, CRC, noncanonical Wnt signalling, p300

## Abstract

Butyrate, a breakdown product of dietary fibre, may in part mediate the ability of a high‐fibre diet to reduce the risk of colorectal cancer (CRC). However, CRC can still develop despite a high‐fibre diet; hence, butyrate resistance may influence colonic tumorigenesis. To model butyrate resistance in vitro, butyrate‐resistant cells were developed and mechanisms identified by which these cells evade the effects of butyrate. These mechanisms can be interpreted in light of the existing literature to further our understanding of butyrate resistance. The current review integrates findings from various studies from my laboratory on butyrate‐resistant cells, in addition to other work in the literature, to present a model of how butyrate‐resistant CRC cells balance different signalling outputs to generate the resistant phenotype. Loss of p300 expression in butyrate resistance allows increased noncanonical Wnt signalling to occur without activating differentiation pathways, AKT/PKB survival signalling is activated, and CBP‐Wnt activity is maintained in the pro‐proliferative range. Further, overexpression of Tcf3 suppresses butyrate‐induced Wnt hyperactivation. Other factors, signalling pathways and modifying influences also affect butyrate sensitivity vs. resistance. Understanding the possible role of butyrate resistance will assist in improving chemopreventive strategies for this disease.

**Abbreviations**
CBP: CREB Binding Protein
CRC: colorectal cancer
HDACi: histone deacetylase inhibitor
KO: knockout
PKC: protein kinase C

## 1 Introduction

Dietary fibre is protective against colorectal cancer (CRC), an effect in part mediated through the histone deacetylase inhibitor (HDACi) butyrate, which is produced by the breakdown of fibre in the colon [1, 2, 3, 4, 5, 6, 7, 8, 9, 10, 11]. Butyrate induces CRC cell cycle arrest, differentiation and/or apoptosis in vitro [1, 2, 12, 13, 14, 15, 16, 17, 18].

Butyrate is present in the in vivo human colonic lumen at mM concentrations and demonstrates the ability to suppress growth and induce apoptosis of CRC cells in vitro [10]. Butyrate is not only produced by colonic bacteria but has been shown to modulate the composition of the gut microbiota in a manner conducive to anti‐carcinogenic activity [19]. Butyrate was shown to induce differentiation and apoptosis of human CRC cells in culture; these traits are shared by other HDACis [12, 13, 14, 15, 16, 17]. The relationship between butyrate, Wnt signalling and CRC cell apoptosis was demonstrated by a direct linear relationship between the degree of Wnt signalling hyperactivation by butyrate and the levels of apoptosis in a spectrum of CRC cell lines [1]. Further, the use of dominant negative Wnt signalling factors confirmed that there is a causal relationship between the ability of butyrate to enhance Wnt activity and to induce apoptosis [1]. In addition, butyrate‐resistant CRC cells displayed suppressed Wnt hyperactivation concomitant with inhibited apoptosis after exposure to butyrate [2]. The protective action of butyrate against the early stages of colonic tumorigenesis was demonstrated by its ability to suppress cell growth to a greater degree in colon adenoma cells [3] as opposed to CRC cells [3].

Most CRCs are initiated by deregulated canonical Wnt signalling [20, 21, 22, 23, 24, 25, 26, 27, 28, 29, 30], and in these CRC cells, HDACis such as butyrate hyperactivate Wnt activity [1, 2, 18]. Hyperactivation of oncogenic signalling can induce apoptosis [31]; thus, my laboratory has shown that increased Wnt signalling, induced by HDACis, is causally linked to the ability of these agents to stimulate CRC cell apoptosis [1, 2]. The ability of butyrate to hyperactivate canonical Wnt signalling and induce CRC cell apoptosis (and growth arrest) may in part explain the protective action of dietary fibre against CRC. Butyrate treatment can also enhance the efficacy of chemotherapy [32] in addition to its known activity for prevention of CRC [33].

Colonic tumorigenesis is optimally promoted by moderate levels of deregulated Wnt signalling [32]. This observation is supported by studies, demonstrating that abnormally elevated levels of canonical Wnt activity, resulting from genetic activation of Wnt signalling, induce apoptosis [34, 35, 36, 37]. These data support our findings that HDAC‐induced hyperactivation of canonical signalling promotes CRC cell apoptosis [1, 2, 18]. Subsequent studies have also demonstrated that genetic and pharmacological activation of Wnt signalling induces apoptosis in non‐colon cells [38, 39, 40], supporting the premise that hyperactivation of oncogenic signalling leads to apoptosis.

In summary, canonical Wnt signalling that is deregulated by mutation results in moderate levels of Wnt activity, which promotes colonic cell proliferation and tumorigenesis. On the other hand, both relatively high and relatively low levels of canonical Wnt signalling activity lead to enhanced CRC cell apoptosis and repressed cell proliferation [1, 2, 18, 40].

CRCs can still develop in individuals with higher levels of dietary fibre intake [6, 7, 8]. This suggests that butyrate resistance may in some cases contribute in part to the process of colonic neoplasia in such individuals. There is currently no definitive direct evidence for butyrate resistance contributing to human colonic carcinogenesis. However, there is a number of lines of indirect evidence supporting the likelihood of butyrate resistance being an important factor in CRC for at least some fraction of patients. First is the data on the protective role of fibre against CRC as well as the known anticancer effects of butyrate, coupled with understanding that while high‐fibre diets may decrease CRC risk, they do not eliminate risk. CRC can still occur with a high‐fibre diet and in the presence of mM concentrations of butyrate known to induce colonic cell growth arrest and apoptosis in vitro [1, 2, 10]. Importantly, there is evidence that resistance to butyrate may be involved in the development of inflammatory bowel disease (IBD) [41], and IBD is a known risk factor for CRC. It is therefore possible that butyrate resistance may influence colon carcinogenesis in non‐IBD patients as well.

In addition, the ability of butyrate to inhibit cell growth is associated with ERK1/2‐c‐myc‐mediated nuclear localization [42] of the cell cycle‐inhibitor p21 [43]; of relevance to potential clinical evidence for butyrate resistance in human patients is the finding that patients with high expression of p21 exhibited a 23% increased five‐year survival rate compared to those with lower p21 expression [42]. Thus, this suggests the possibility that CRCs that are more butyrate resistant exhibit less p21 expression and nuclear p21, leading to decreased patient survival. In addition, four butyrate metabolism‐related genes (*FN1, SERPINE1, THBS2* and *COMP*) were associated with high risk for CRC in human patients [44]. This also suggests a link between butyrate resistance and colonic tumorigenesis. Higher levels of E‐cadherin were found to be associated with increased five‐year survival in CRC patients [45]; butyrate is known to upregulate E‐cadherin expression [46]. Therefore, butyrate resistance may decrease E‐cadherin expression, lessening the protective effects of butyrate and worsening outcomes for CRC.

Butyrate levels were found to be higher in CRC patients and in patients at risk for CRC [47], which further suggests the possibility of butyrate resistance given that agent's known potent anticancer effects in vitro. However, some studies have also shown lower butyrate in CRC, consistent with the idea that the microbiome is important for translating diet into particular butyrate levels [47]. Despite these inconsistencies, it is clear that CRC can develop in the presence of butyrate; hence, butyrate resistance is a likely in vivo contributor to colon tumorigenesis. The totality of the evidence strongly suggests a role for butyrate resistance in at least some cases of CRC. More conclusive evidence for butyrate resistance in human CRC patients would likely require ex vivo studies of cells obtained from removed CRCs, coupled to information on patient dietary habits and intra‐colonic levels of butyrate. It is hoped that the present review will stimulate further research into this area.

Butyrate resistance can be modelled in vitro by the production of butyrate‐resistant CRC cell lines, typically generated via selection of cell growth in the presence of increasing concentrations of the agent. CRC cells resistant to 3.2 mM butyrate exhibited increased expression of genes related to butyrate influx and drug efflux, as well as chemoresistance to agents unrelated to butyrate [48]. Another study produced a butyrate‐resistant human colon carcinoma cell line and demonstrated that the butyrate‐resistant cells were apoptosis resistant and displayed enhanced stress survival [49].

Altered Wnt signalling may be involved in butyrate resistance. To investigate this possibility, my laboratory developed [2] a butyrate‐resistant CRC cell line (HCT‐R) from butyrate‐sensitive HCT‐116 CRC cells that were exposed to increasing levels of butyrate (up to 5 mM, a physiologically relevant concentration that induces CRC cell apoptosis and inhibits cell growth). HCT‐R cells exposed to butyrate exhibit suppressed canonical Wnt signalling hyperactivation, lower levels of apoptosis and increased cell growth compared to parental HCT‐116 cells [2]. In addition, HCT‐R cells are cross‐resistant to other clinically relevant and structurally distinct HDACis, some of which have been evaluated as part of combinatorial therapy against metastatic CRC 51]. HCT‐R cells treated with these HDACis exhibit suppressed canonical Wnt activity and apoptosis, and increased cell growth, similar to what is observed when these cells are exposed to butyrate [2].

The mechanisms whereby butyrate/HDACi resistance develops in CRC cells were analysed. One study identified overexpression of the *TCF7L1* gene as a contributing factor to butyrate resistance [50]. *TCF7L1* overexpression results in high levels of the Tcf3 transcription factor, which inhibits canonical Wnt activity, thus inhibiting Wnt hyperactivation. Another mechanism whereby CRC cells develop resistance HDACis is through increased noncanonical as opposed to canonical Wnt signalling [51]. Thus, butyrate‐resistant cells upregulate expression of Wnt ligands such as WNT5A; these ligands are channelled to induce noncanonical Wnt signalling via the concomitant upregulation of the ROR2 receptor [51]. Enhanced noncanonical Wnt signalling is causally associated with suppressed butyrate‐induced upregulation of canonical Wnt signalling and greater clonal growth; this was demonstrated through ROR2 knockdown in butyrate‐resistant HCT‐R cells and overexpression of WNT5A in butyrate‐sensitive HCT‐116 cells [51]. These effects are in part mediated by activation of AKT/PKB signalling, which is itself mediated by WNT5A and ROR2; thus, inhibitors of pAKT can partially resensitize resistant cells to HDACis [51]. HCT‐R cells also display a degree of resistance to the non‐HDACi chemotherapeutic drug 5‐fluorouracil, suggesting that the switch from canonical to noncanonical Wnt signalling induces a more general drug‐resistant phenotype [51].

These findings only partially explain the HDACi‐resistant phenotype. Further evaluation of gene expression data identified p300 as a factor whose expression is downregulated in HCT‐R cells [50]. The histone acetylases (HATs) CBP and p300 are important in colonic tumorigenesis, and the association between beta‐catenin and CBP or p300 influences Wnt signalling [52, 53, 54, 55, 56, 57, 58, 59, 60, 61, 62]. CBP and p300 compete for binding to beta‐catenin [52]. Thus, CBP‐Wnt and p300‐Wnt activities are negatively correlated; increased CBP‐Wnt activity leads to decreased p300‐Wnt activity and *vice versa*. Canonical Wnt activity mediated by CBP promotes cancer cell proliferation, stemness and expression of the anti‐apoptotic factor survivin; in contrast, canonical Wnt signalling mediated by p300 promotes differentiation [52, 53, 54, 55, 56, 57, 58, 59, 60, 61, 62] and may mediate sensitivity to butyrate [63, 64, 65, 66, 67, 68, 69, 70, 71, 72, 73, 74, 75, 76, 77, 78, 79, 80, 81, 82]. Hyperactivation of either or both of these Wnt pathways may promote apoptosis. CBP‐Wnt activity tends to predominate in CRC cells [63, 64, 65, 69, 82], and while CBP‐Wnt activity is typically pro‐proliferative (and anti‐apoptotic due to induction of survivin) [52, 53, 54, 55, 56, 57, 58, 59, 60, 61], abnormal hyperactivation of this signalling by HDACis such as butyrate triggers apoptosis [1, 2, 11, 12, 13, 14, 15, 16, 17, 18, 63, 64, 65, 69, 82]. Hyperactivation of p300‐Wnt signalling, particularly in the context of inhibition of CBP‐Wnt signalling, also stimulates CRC cell apoptosis [63, 64, 65, 69, 82].

The association between CBP and Wnt signalling is typically investigated utilising the small molecule inhibitor ICG‐001 that binds to CBP but not to p300, repressing CBP‐Wnt activity and maintaining or increasing p300‐Wnt activity [52]. Thus, treatment of CRC cells with ICG‐001 downregulates canonical Wnt transcriptional activity through targeting the CBP‐Wnt component of that signalling in neoplastic colonic cells [52]. In addition, apoptosis of CRC cells is typically induced by treatment with ICG‐001 [52]. Findings from my laboratory documented cell type‐specific effects of combinatorial treatment of CRC cells with butyrate and ICG‐001 [63, 64, 65, 69, 82]. Some CRC cell lines exhibited interference between these agents in their ability to induce apoptosis, while in other lines there was no interference, and in certain cases an additive effect on apoptosis was observed [63, 64, 65, 69, 82]. Furthermore, our findings, as well as those of the literature, demonstrated that *EP300* (p300) expression influences butyrate sensitivity vs. resistance [63, 64, 65, 66, 67, 68, 69, 70, 71, 72, 73, 74, 75, 76, 77, 78, 79, 80, 81, 82]. HCT‐R cells do not express p300, and HCT‐116 cells that are *EP300*/p300 knockout (KO) exhibit partial butyrate resistance; further, restoration of p300 expression in the KO cells resensitises them to the effects of butyrate [63, 64, 65, 69, 82].

Mechanisms underlying butyrate resistance in CRC cells may exhibit cross‐talk; thus, differential use of CBP and p300 can contribute to choices of canonical and noncanonical Wnt signalling [83] that can affect drug resistance and other aspects of cell phenotype. Canonical Wnt signalling tends to induce CBP‐Wnt activity that is pro‐proliferative; however, increased noncanonical Wnt signalling can activate kinases (e.g., protein kinase C [PKC]) that phosphorylate p300, increasing the affinity of p300 for beta‐catenin [83]. Increased p300‐beta‐catenin binding and p300‐Wnt activity leads to decreased CBP‐beta‐catenin binding and CBP‐Wnt activity, favouring differentiation and apoptosis over proliferation [83].

Altered expression of CBP or p300 can modulate the relative sensitivity of cells to butyrate by switching between (a) a more proliferative CBP‐mediated Wnt activity promoted by canonical Wnt signals (e.g., WNT3a) or (b) a more differentiating p300‐mediated Wnt activity promoted by noncanonical Wnt signals (e.g., WNT5a) [83]. The choice between these pathways can also be pharmacologically modulated; thus, the CBP‐Wnt inhibitor ICG‐001 (or activation of PKC) would favour the p300‐mediated pathway, while inhibitors of p300‐beta‐catenin association (or of PKC activity) would tend to favour the CBP‐mediated pathway [83]. Note that the ability of the cell to switch between these pathways depends upon the expression of the relevant HATs. For example, suppressed expression of p300, as observed in butyrate‐resistant HCT‐R cells [63, 64, 65, 69, 82], means that activation of noncanonical Wnt activity would not be able to favour a p300‐mediated signalling pathway and consequent differentiation.

## 2 A Model of Wnt Signalling and p300 for Butyrate Resistance in CRC

**FIGURE 1:**
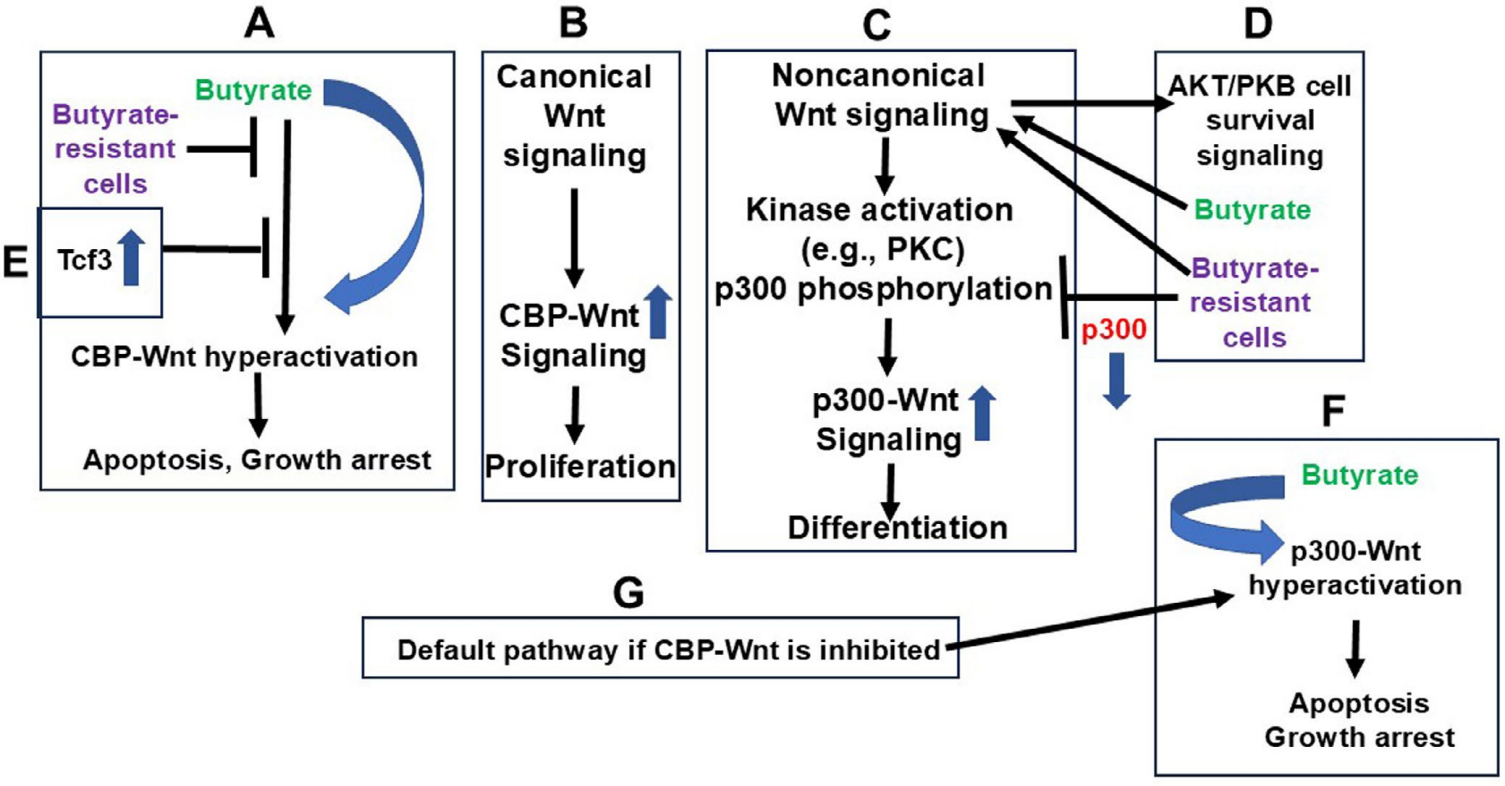
Wnt signalling and CBP/p300 in butyrate resistance of colonic cells. I propose the following mechanism of butyrate resistance as modelled by HCT‐R cells. (A) Butyrate promotes CBP‐Wnt hyperactivation leading to apoptosis and/or growth arrest; this is inhibited in butyrate‐resistant cells. (B) (C) Canonical Wnt signalling, particularly in CRC, promotes CBP‐Wnt activity that is associated with cell proliferation. Noncanonical Wnt signalling normally activates kinases that phosphorylate p300, increasing p300‐beta‐catenin association and p300‐Wnt signalling at the expense of CBP‐Wnt activity. The result of this increased p300 signalling would be differentiation. Both CBP‐ and p300‐mediated Wnt signalling can be hyperactivated by butyrate (and other HDACis), promoting apoptosis and growth arrest. (D) Butyrate‐resistant HCT‐R cells exhibit several changes that influence this scenario. Suppressed expression of p300 occurs, so that the p300‐mediated Wnt signalling branch is non‐existent. (E) Overexpression of *TCF7L1*, resulting in high levels of its product Tcf3, inhibits the remaining CBP‐Wnt activity, suppressing Wnt hyperactivation by butyrate and consequent apoptosis and growth arrest. However, noncanonical Wnt signalling, increased in HCT‐R cells, enhances AKT/PKB cell survival signalling, and this can be stimulated by butyrate. The net result of all these effects is continued cell proliferation in the presence of levels of butyrate that would induce apoptosis in butyrate‐sensitive cell lines, such as HCT‐116. The loss of p300 expression in HCT‐R cells, combined with the overexpression of *TCF7L1*, is key here, for if the p300 was present, and *TCF7L1* not overexpressed, then (F) p300‐Wnt hyperactivation, which is the default pathway if (G) CBP‐Wnt activity is inhibited, would likely allow for apoptosis even in the presence of AKT/PKB activity. Thus, the interaction of these signalling pathways decides the outcome of switching between canonical and noncanonical Wnt signalling in butyrate‐resistant CRC cells.

Based on the available findings in the literature, including those from my laboratory, a model for butyrate resistance in CRC is proposed (Figure 1). Thus, the loss of p300 expression in the context of butyrate resistance in HCT‐R cells allows for increased noncanonical Wnt signalling without activating the p300‐mediated pathway. Instead, AKT/PKB survival signalling is activated, and CBP‐Wnt activity is maintained in the pro‐proliferative range since the Tcf3 factor inhibits pro‐apoptotic Wnt hyperactivation. In this manner, an integrated series of events occurs that are required for the development of a fully butyrate‐resistant CRC cell phenotype.

This model is a combinatorial derivation of previous work in this field, which is outlined in the Introduction. By bringing together separate pieces of the puzzle of butyrate resistance, a novel and more complete synthesis is thus generated that can provide a more comprehensive understanding of the phenomenon and its scientific and clinical implications. Thus, this review introduces an integrated model of how CRC cells can balance different signalling outputs to generate the butyrate resistant phenotype.

Previous attempts to overcome butyrate resistance may have failed because of a lack of understanding that multiple interacting pathways, including both canonical and non‐canonical Wnt signalling, are involved. In addition, the relevance of CBP‐Wnt vs. p300‐Wnt signalling to the question of butyrate resistance was unknown until recently. Thus, the present model is novel in that it does not depend on a single explanation or single pathway to explain butyrate resistance, but at the same time, it proposes that a limited number of signalling pathways are common to many, if not most, examples of butyrate resistance. In other words, the solution is multifactorial, but it is also knowable and testable.

Interactions between these pathways may explain why ICG‐001 and butyrate cotreatment interferes with the induction of apoptosis in HCT‐R cells compared to treatment with ICG‐001 alone, while no interference occurs in HCT‐116 cells [63, 64]. In butyrate‐sensitive HCT‐116 cells, both CBP and p300 are expressed, so inhibition of CBP–Wnt activity by ICG‐001 maintains p300–Wnt activity, which can be hyperactivated by butyrate, allowing for apoptosis (Figure 1). Thus, p300 knockdown with siRNA reduces the ability of butyrate to hyperactivate Wnt signalling [64], demonstrating that Wnt hyperactivation can be mediated by p300 just as it can be mediated by CBP [61]. It is important to note that the levels of butyrate‐induced Wnt activity are reduced but not eliminated by ICG‐001/butyrate cotreatment [63], suggesting that (a) Wnt hyperactivation is typically mediated by CBP in CRC cells, and (b) Wnt hyperactivation can also be mediated by non‐CBP (e.g., p300) pathways. In addition, data from p300 knockout (KO) HCT‐116 cell lines support the importance of p300 in butyrate resistance. Thus, the F5 (but not D10) p300 KO HCT‐116 CRC cell line exhibits reduced Wnt hyperactivation by butyrate, and this was rescued by restoration of p300 expression [69]. All these data support the idea that Wnt hyperactivation by butyrate has both CBP‐ and p300‐mediated components.

In HCT‐R cells, suppression of p300 expression eliminates p300‐Wnt activity. Treatment with ICG‐001 alone stimulates apoptosis in this cell line [64] by repressing the remaining CBP‐Wnt activity and inhibiting expression of the anti‐apoptotic factor survivin. Upon cotreatment with butyrate and ICG‐001 (Figure 1), there is no alternative p300‐Wnt hyperactivation pathway to induce apoptosis and growth arrest. Instead, AKT/PKB survival signalling is activated by butyrate [51]. Thus, the action of ICG‐001 alone in stimulating apoptosis in HCT‐R cells is counteracted by increased AKT/PKB survival signalling induced by butyrate.

At the same time, the absence of p300 prevents the induction of apoptosis and growth arrest via hyperactivated p300‐Wnt signalling. Butyrate cannot hyperactivate CBP‐Wnt signalling in this scenario because of the inhibitory action of ICG‐001. What is expected to occur in HCT‐R cells treated with butyrate alone? Even in the absence of ICG‐001, Tcf3 overexpression in HCT‐R cells would repress CBP‐Wnt hyperactivation and therefore inhibit apoptosis and growth arrest (Figure 1). All these events can occur because of the downregulation of p300 in this cell line.

It is also important to note that *TCF7L1* overexpression alone cannot fully explain butyrate resistance in HCT‐R cells, since siRNA knockdown of *TCF7L1* only partially restores responsiveness to butyrate [50]. In addition, while exogenous overexpression of *TCF7L1* in HCT‐116 cells can suppress canonical Wnt activity, it also represses cell growth. Therefore, *TCF7L1*‐overexpressing HCT‐116 cells do not proliferate in the presence of butyrate (as do HCT‐R cells). In contrast, HCT‐R cells that couple *TCF7L1* overexpression [50] with loss of p300 expression [53] and activation of AKT/PKB signalling [51] upregulate cell cycle progression factors (cyclins, cyclin‐dependent kinases and CDC25A,B,C) [50, 51]. Thus, in HCT‐R cells (a) increased expression of the *TCF7L1* product Tcf3 reduces CBP‐Wnt hyperactivation; and (b) activation of cell survival signalling and induction of cell cycle factors, and maintenance of moderate pro‐proliferative levels of CBP‐Wnt activity, allow for cell growth in the presence of butyrate and of overexpressed Tcf3 protein. Thus, while *TCF7L1*/Tcf3 prevents CBP‐Wnt hyperactivation in HCT‐R cells, it is the additional loss of p300 and the activation of AKT/PKB signalling that are responsible for the full butyrate‐resistant phenotype.

Thus, available findings suggest that for some CRCs it is the loss of p300 that ‘tips the balance’ in favour of cell survival and butyrate resistance. It is interesting to note that HCT‐116 cells, which express p300, also exhibit upregulation of pAKT after butyrate treatment [51], yet these cells still respond to butyrate with apoptosis. Thus, the current model posits that the loss of p300 in HCT‐R cells enables increased AKT/PKB signalling to stimulate proliferation despite these cells being exposed to butyrate. The fundamental importance of p300 is underscored by the finding that loss of p300 expression alone is sufficient to induce a significant degree of butyrate resistance in the HCT‐116 line, which can be reversed upon restoration of p300 expression [69].

## 3 To Summarise the Situation in HCT‐R Cells

1. Overexpression of *TCF7L1* reduces CBP‐Wnt hyperactivation by butyrate, preventing induction of apoptosis and growth arrest. Thus, moderate levels of CBP‐Wnt activity are maintained to drive cell proliferation.
2. The overexpression of *TCF7L1* itself does not repress cell growth, as it does in butyrate‐sensitive cells, since HCT‐R cells also overexpress a variety of cell cycle progression factors.
3. The loss of p300 decreases the probability that significant Wnt hyperactivation by butyrate occurs. Downregulation of p300 also ensures that increased noncanonical Wnt activity drives AKT/PKB cell survival signalling, rather than p300‐Wnt activity (that would have promoted differentiation/apoptosis).
4. Increased cell survival signalling may be responsible for the upregulated expression of cell cycle factors.

Therefore, the loss of p300 is the crucial step tying these different pathways together to produce butyrate resistance in HCT‐R cells.

Different butyrate‐resistant CRC cell lines, or primary tumours, would not necessarily exhibit the same specific changes as does the HCT‐R cell line, but I posit that the basic alterations would be functionally similar (assuming that the CRC in question is positive for Wnt deregulation). Thus, it is reasonable to expect that some mechanism, akin to *TCF7L1* overexpression, would repress hyperactivation of canonical Wnt signalling. It is also likely that a type of cell survival signalling would be activated. Further, given the importance of CBP‐ vs. p300‐mediated signalling pathways in CRC, it is also likely that alteration in these pathways would occur. Downregulation of *EP300* may be a common factor in many cases of butyrate resistance in CRC. HCT‐116 cells normally exhibit association between p300 and beta‐catenin [52], so butyrate‐resistant HCT‐116 cells (i.e., the HCT‐R line) downregulate expression of p300.

On the other hand, SW620 cells normally already exhibit minimal to no association of p300 with beta‐catenin [64], so it is possible that butyrate resistance in SW620 cells would not require downregulation of p300 expression; for example, a butyrate‐resistant SW620 cell line generated in my laboratory still expresses p300 [84, 85]. Thus, CRC cells already lacking p300‐Wnt activity would not require the loss of this activity to develop butyrate resistance, as they naturally lack this component of the butyrate response. Thus, every CRC cell line studied in my laboratory that is fully (HCT‐R and SW620‐R) or partially (*EP300* knockout HCT‐116 cells) resistant to butyrate lacks p300‐Wnt activity, either because of experimental procedures (the HCT‐116‐based lines) or because the parental cells already lack this activity (SW620). The situation in SW620 cells is further discussed below, with respect to testing the model.

## 4 Extending the Model of Butyrate Resistance: The Role of Other Factors

Other signalling pathways, factors and modifying influences also influence butyrate sensitivity vs. resistance. The following describes some findings in this field.

MAPK activity stimulates CBP/p300 transactivation, likely through phosphorylation of these factors [86]. Indeed, CBP/p300 integrates many signalling inputs and these may be involved in mediating the butyrate‐resistant phenotype in CRC. MAPK/ERK2 signalling also mediates effects of butyrate on CRC cells through endocan expression [87], and likely through other signalling cross‐talk as well. The ERK1/2‐c‐myc signalling cascade also mediates effects of butyrate on suppressing cell growth via enhanced nuclear localisation of p21 [42]. Induction of butyrate resistance in CRC cells results in downregulation of AMPK signalling and upregulation of the mTOR pathway, causing a protective form of autophagy; butyrate sensitivity can be restored via AMPK activation and repressed mTOR [88]. Obviously, other factors and pathways, beyond the scope of the present review, also influence butyrate action. Indeed, even when focusing on Wnt‐specific gene expression, exposure to butyrate induces wide‐ranging changes in gene expression in CRC cells [76], and the role of these changes in butyrate resistance remains to be fully explored.

There is evidence of cell‐type specificity in the effects of butyrate in that inhibition of cell growth associates with increased aggressiveness, as measured by tumour formation and metastasis in an animal model [42]. If this correlates to the human situation, butyrate may be most effective against the more aggressively growing neoplastic colonic cells. FAK1/Src1/E‐cadherin signalling is also important for cell‐type effects of butyrate treatment, and higher levels of E‐cadherin have been clinically shown to be associated with a 13% increase in five‐year survival in CRC [45].

Further, number of molecular subtypes of CRC have been identified, each with different sets of driver gene mutations and activated signalling pathways, and some characteristics of these tumours may influence sensitivity vs. resistance to butyrate [89]. Thus, Wnt signalling is important for the CMS2 type, and given the data from our laboratory linking hyperactivation of Wnt signalling to response to butyrate, CMS2 tumours may be particularly sensitive to butyrate, but, on the other hand, may also be prone to develop butyrate resistance as a mechanism to evade this innate sensitivity. MAPK signalling is important in CMS2 and CMS3 subtypes; to the extent MAPK signalling influences butyrate resistance via effects on CBP/p300 signalling, this subtype may also demonstrate a differential propensity or butyrate resistance. There needs to be a more targeted examination of how butyrate‐relevant signalling pathways differ between these subtypes and how this variation affects the tendency of these types to develop resistance when exposed to butyrate. For example, the contradictory findings with respect to the effects of butyrate on CRC progression, which some call ‘the butyrate paradox’, and this may be due the differentiation state of the colonic epithelium, emphasising the importance of cell‐type effect and CRC sub‐types with respect to butyrate activity [90].

**FIGURE 2:**
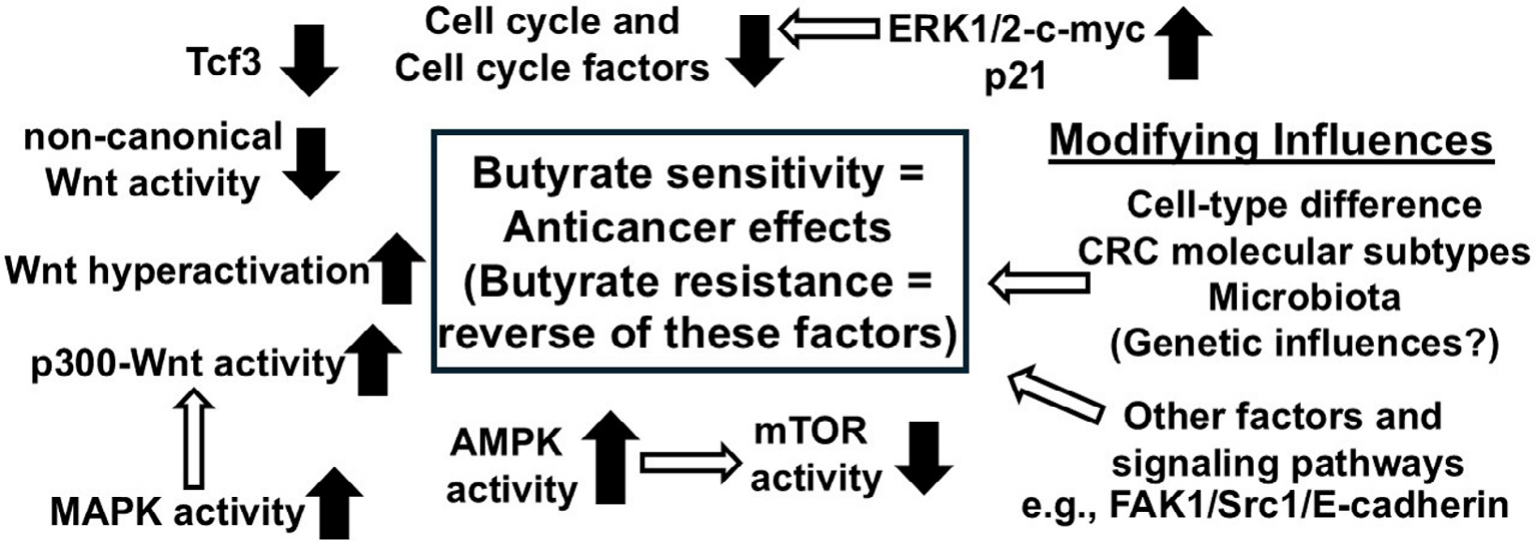
A model of butyrate action and butyrate resistance in CRC. Signalling pathways, factors, and modifying influences reviewed in the current paper that can affect butyrate's anticancer activity against CRC are shown. Solid arrows represent increased or decreased levels of activity that would lead to increased butyrate sensitivity, activity and enhanced anticancer action. The situation for each factor and pathway would be reversed in butyrate reissuance. Empty arrows represent possible interactions between pathways. Important modifying influences are shown at right. The extent to which each factor or pathway affects butyrate activity is affected by cell‐type effects as well as CRC molecular subtypes. Other factors and pathways not specifically designated, some heretofore unknown, may also influence butyrate sensitivity vs. resistance.

Integrating these additional factors, pathways and influences, with the more Wnt‐specific effects described in Figure 1 provides a more comprehensive model of the factors influencing butyrate's anticancer activity reviewed in this manuscript; reversal of these influences would result in butyrate resistance (Figure 2).

Butyrate, and by extension a high‐fibre diet that can result in elevated colonic butyrate, has the potential as an anticancer agent against CRC, as reviewed in the current paper. Given the possible importance of butyrate action, resistance to this agent must be considered. Butyrate resistance likely plays a role in colonic tumorigenesis, particularly in the context of a high‐fibre diet. This effect is expected to be greatest for right‐sided colon tumours, since levels of butyrate are highest in the right (proximal) colon [66], and colonic butyrate levels are considered a key factor in CRC prevention [40]. Overexpression of nuclear p300 is associated with a more favourable prognosis in colon, but not rectal, cancer [68]. The difference between the colon and rectum is important in that butyrate resistance is expected to occur in areas where levels of butyrate are higher, e.g., the more proximal portions of the colon. Thus, given the possibility of butyrate resistance influencing carcinogenesis, suppressed expression of p300 is expected to lead to a worse prognosis for cancers that develop where butyrate levels are higher (colon vs. rectum).

## 5 Discussion

The current review integrates information from the literature combined with data from my laboratory to form a model that brings together a number of previously disparate theories of butyrate resistance to form a more comprehensive whole. The current manuscript should serve the epistemological purpose of stimulating interest in this topic, to prompt future experimental investigation of these questions, as well as to further our understanding of butyrate resistance in the process of colonic tumourigenesis.

Investigating the models proposed in the current manuscript will aid in our understanding of the mechanisms driving butyrate/HDACi resistance, which can assist in developing approaches [91, 92, 93] to prevent and/or reverse resistance. Follow‐up studies can focus on integrating the model outlined here with the possible roles of Sam68 and Pygo2 in mediating butyrate resistance [94], which have possible therapeutic significance [94, 95, 96, 97, 98].

Such approaches would enhance the utility of dietary fibre as a preventive factor against CRC and improve the current limited efficacy [64, 65, 66] of HDACis as anti‐CRC therapeutic agents. The findings of the proposed studies could also inform regarding optimal anti‐cancer approaches involving modulation of CBP and p300 [97].

Complicating the butyrate story are recent findings that butyrate may increase chemoresistance in CRC [99]. Thus, the anticancer efficacy of butyrate may be more focused on prevention, with a variety of positive and negative effects after a tumour is established. This underscores the need for more investigation into the action of butyrate in colonic cells, including the development of butyrate resistance.

## 6 Permission to Reproduce Material From Other Sources

Not applicable.

## 7 Clinical Trial Registration

Not applicable.

## Author Contributions

**Michael Bordonaro:** conceptualization (equal), supervision (equal), visualization (equal), writing – original draft (equal), writing – review and editing (equal).

## Ethics Statement

The author has nothing to report.

## Consent

The author has nothing to report.

## Conflicts of Interest

The author declares no conflicts of interest.
